# Motivation and Intelligence Drive Auditory Perceptual Learning

**DOI:** 10.1371/journal.pone.0009816

**Published:** 2010-03-23

**Authors:** Sygal Amitay, Lorna Halliday, Jenny Taylor, Ediz Sohoglu, David R. Moore

**Affiliations:** Medical Research Council Institute of Hearing Research, Nottingham, United Kingdom; Ecole Polytechnique Federale de Lausanne, Switzerland

## Abstract

**Background:**

Although feedback on performance is generally thought to promote perceptual learning, the role and necessity of feedback remain unclear. We investigated the effect of providing varying amounts of positive feedback while listeners attempted to discriminate between three identical tones on learning frequency discrimination.

**Methodology/Principal Findings:**

Using this novel procedure, the feedback was meaningless and random in relation to the listeners' responses, but the amount of feedback provided (or lack thereof) affected learning. We found that a group of listeners who received positive feedback on 10% of the trials improved their performance on the task (learned), while other groups provided either with excess (90%) or with no feedback did not learn. Superimposed on these group data, however, individual listeners showed other systematic changes of performance. In particular, those with lower non-verbal IQ who trained in the no feedback condition performed more poorly after training.

**Conclusions/Significance:**

This pattern of results cannot be accounted for by learning models that ascribe an external teacher role to feedback. We suggest, instead, that feedback is used to monitor performance on the task in relation to its perceived difficulty, and that listeners who learn without the benefit of feedback are adept at self-monitoring of performance, a trait that also supports better performance on non-verbal IQ tests. These results show that ‘perceptual’ learning is strongly influenced by top-down processes of motivation and intelligence.

## Introduction

Practice may not “make perfect”, but it can certainly improve skills and abilities, including the ability to detect or discriminate a variety of sensory stimuli, a process known as perceptual learning. Knowledge of results, or information on task performance would seem to be a necessary component for learning. However, in perceptual learning the importance of, or even the need for performance feedback is a controversial topic [1]–[11]. The issue of performance feedback is also important from an applied perspective: the numerous commercially available packages using perceptual training to enhance auditory, visual and cognitive performance in impaired [12]–[14] and elderly [15] populations employ performance feedback as a matter of course. Understanding the effect(s) of feedback on learning is therefore of practical concern in optimizing training programmes that are of potential benefit to many.

Under some circumstances, feedback on performance does not appear to be necessary for successful learning [4]–[9]. Such learning appears to occur when the training task includes sufficiently easy trials, where success is obvious to the trainee. Yet even in conditions where learning can occur without feedback, providing feedback can substantially facilitate the learning in terms of speed and final performance [1], [2], [10]. Thus, even if feedback is not necessary for learning, it can still be beneficial. In other cases learning did not occur unless feedback was provided [7], [16]. The success of learning without feedback appears to depend on the difficulty of the training task [17], with easy tasks enabling learning without feedback, more difficult tasks benefitting from feedback, and the most difficult tasks requiring feedback for learning. This explanation implicitly assumes that feedback, when used, is used in an immediate fashion on a trial-by-trial basis to correct the learning mechanism, and is supported by findings that learning did not occur when the feedback was uncorrelated with the participants' responses [10].

We have previously reported robust learning on an ‘impossible’ auditory frequency discrimination training task that used identical stimuli [18]. The feedback provided was meaningless; it was completely uninformative about performance and uncorrelated with the listeners' responses. Yet the degree of learning was comparable to that experienced by listeners in a number of other conditions where there was an actual difference between the target and comparison tones and the feedback was dependent on listeners' responses. Two possible explanations of these results are that, firstly, the small number of trials used to assess performance prior to training may have been sufficient to bootstrap the learning – a sort of ‘eureka effect’ [19]. This possibility is unlikely; it has been shown [16] that including easy trials among difficult trials does not promote learning when no external feedback is provided. Secondly, the (random and meaningless) feedback may have been ignored, and learning proceeded without it. However, it would be hard to reconcile learning with no feedback on an impossible task with previous demonstrations that difficult tasks required feedback for learning. Based on previous studies, no learning should have occurred in our study, because the task was impossibly difficult and the feedback was useless as a correcting teacher.

Two other explanations that can reconcile our findings [18] with previous findings both require us to abandon the view of externally provided feedback being used for immediate error correction. Firstly, it is possible that listeners ignored the false feedback we provided, and relied instead on internally generated feedback. While it is easy to see how easily discriminable stimuli can generate internal feedback on response correctness, it is not intuitively obvious how attempting to discriminate identical stimuli can do so. However, internal (neural) noise [20], [21] was theoretically shown to be sufficient to engender a percept of frequency differences between the identical tones [22]. This derivation reinforces the anecdotal, subjective perception of pitch differences when performing the task. Listeners may thus use the percept of a different sound to generate internal feedback that supports learning on this impossible task. The second explanation rejects the idea that feedback (either external or internal) provides immediate, trial-by-trial correction. Instead, feedback may be used to motivate, to keep participants engaged and on task. Receiving positive feedback (praise) may encourage participants that they are doing well.

In the study reported here we explored the effect of positive performance feedback on learning an impossible auditory frequency discrimination task. Positive feedback was provided randomly on either 10% or 90% of the trials, and compared with a no-feedback condition and previous data [18] based on 33% positive feedback. If learning depends on internal feedback and the external feedback is ignored, there should be no difference in learning between these conditions; the presence and quantity of feedback should be irrelevant. However, if feedback acts as a motivator, the more positive feedback provided the more motivated participants should be, and hence we could expect to see more learning.

## Results

### Feedback affects group performance

**Figure 1:**
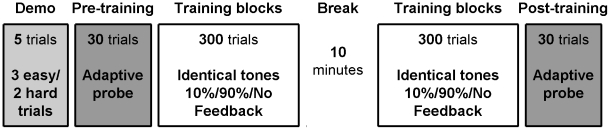
Summary of the experimental design. The experimental procedure consisted of a pre-test that included a short (5-trial) demonstration to familiarize listeners with the task followed by a short probe to assess the DLF, lasting 30 trials [41]. The training phase consisted of 600 trials delivered in 100-trial blocks, with a 10-minute break after the first 3 blocks (300 trials). Groups were trained either with random feedback provided on 10% of trials, 90% or no feedback (NF). Finally, a second probe was administered to assess DLFs following the training.

**Figure 2:**
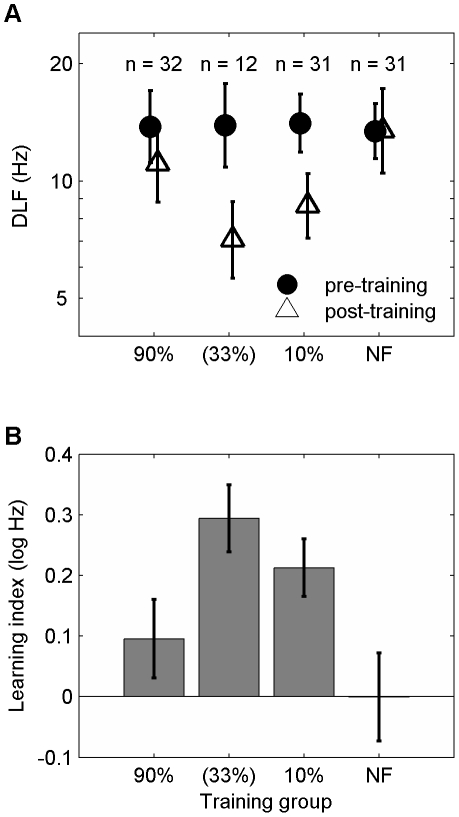
Improvement in Frequency discrimination depends on the type of feedback provided. (A) Mean Frequency discrimination thresholds (DLFs) for the pre- and post-training probes in the three experimental groups (90%, 10% and NF), and the 33% group data adapted from [18] displayed for comparison (but not included in the statistical analysis). Data are shown as mean +/− SEM. (B) Mean learning data (+/− SEM), calculated as the difference in pre- and post-training DLFs. Learning was significant only in the 10% group (p<0.001).

We investigated perceptual learning on an auditory discrimination task using a novel design that allowed us to manipulate the amount of feedback independent of other aspects of the training task. Pure tone frequency discrimination (FD) was measured before and after FD training (Figure 1) where the tones to be discriminated were identical [18]. Two groups of listeners received positive feedback (‘correct’ responses) on 90% or 10% of trials, respectively, while a third group received no feedback (NF). Groups were matched for initial FD ability (Figure 2A) and compared with another group receiving 33% positive feedback (n = 12), collected as part of another study [18]. Following training, FD thresholds (‘difference limen for frequency’ – DLF) improved in some groups but not others, indicating that feedback is an important factor in auditory learning. Significant differences in learning (Figure 2B) were found between the three groups of this study (Repeated-measures ANOVA: F(2,88) = 3.2; p = 0.044), with significant learning observed only in the 10% group (one-sample t-test: p<0.001).

### Individual differences in the use of feedback for learning

**Figure 3:**
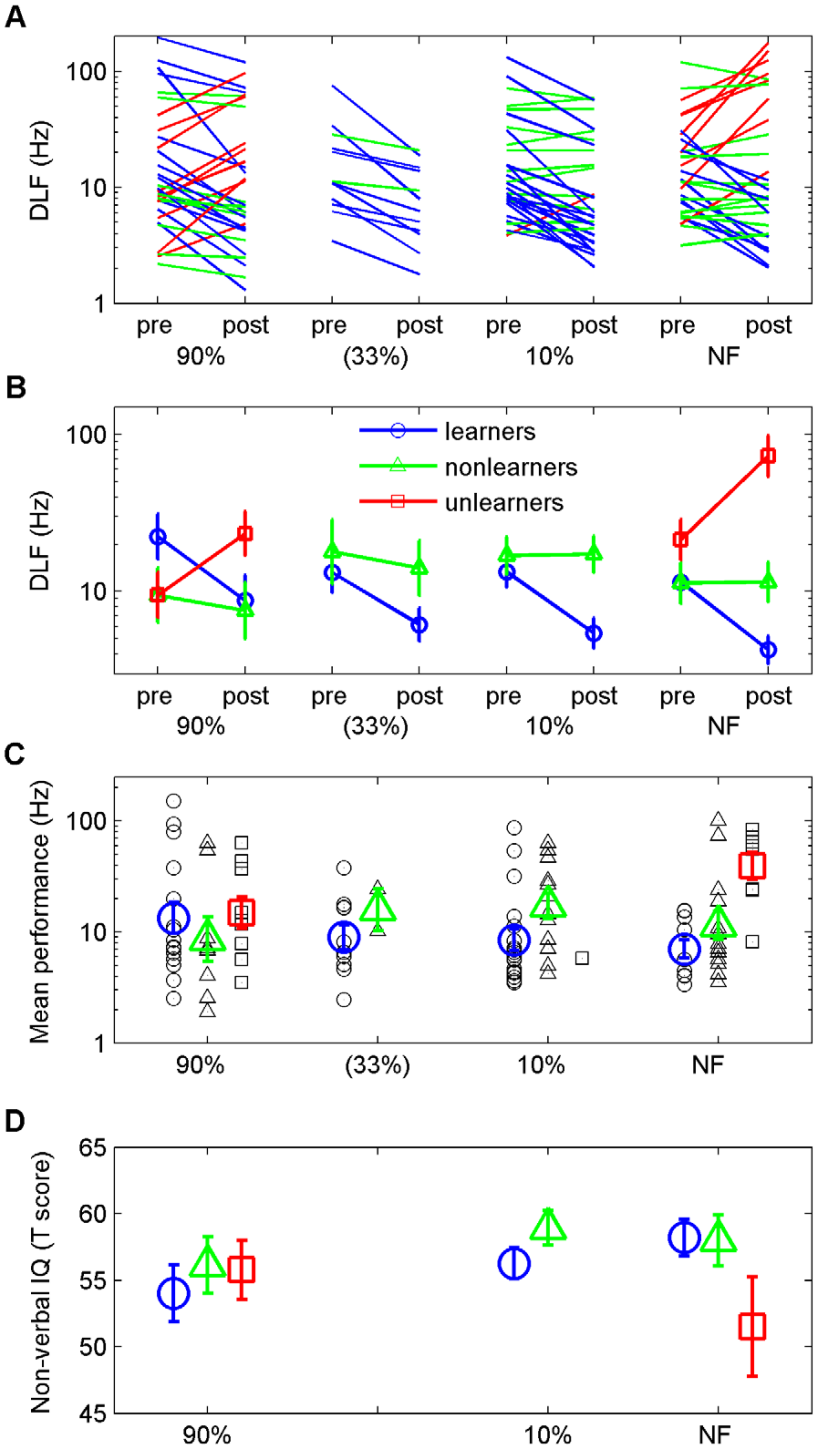
Individual and learning-subgroup data. (A) Individual DLFs on the pre- and post-training probes. Listeners who showed no change in DLFs (‘non-learners’; the change in DLF was less than a factor of √2, which was the step size of the adaptive staircase) are marked in green, those who showed learning (‘learners’) are marked in blue, and those who showed worse performance on the post-test compared to the pre-test (‘unlearners’) are marked in red. The 33% group are included in the figure for comparison but excluded from statistical analysis. (B) Group means of pre- and post-test DLFs by learning subgroup. (C) Average performance (mean of pre- and post-training DLFs) displayed by group and subgroup. (D) Standardized (T) score on the WASI [44] Matrices subtest used to assess non-verbal IQ. All data are represented as means +/− SEM.

Listeners in each training group were divided into three learning subgroups (Figure 3A); learners (reduced DLF), unlearners (increased DLF), and non-learners (DLF changed by <√2). Mean DLFs by subgroup are shown in Figure 3B. In the groups that showed learning (10% and 33%), only a single unlearner (of n = 43) was found. The proportion of unlearners was much higher in the 90% and NF groups (9 of 32 and 8 of 31, respectively). A significant association was found between feedback group and learning subgroup (χ^2^(4) = 9.9; p = 0.041).

Although learning was not found in either the 90% or NF groups (Figure 2B), and the mean pre-training DLF did not differ between these groups (Fig. 2A), the pattern of performance based on learning subgroup did differ between these two groups (Figure 3B). No difference was found between the mean performance (average of pre- and post-training DLFs; Figure 3C) of the three groups of this study (F(2,82) = 2.2, p = 0.12). However, learning subgroups did differ (F(2,82) = 3.5, p = 0.034) on this measure, and the interaction between training group and learning subgroup was significant (F(3,82) = 3.4, p = 0.021). Analysis of this interaction (one-way ANOVA) showed that mean performance differed between training groups only for the unlearners (F(1,15) = 5.03, p = 0.040). Unlearners in the NF group had poorer mean performance (higher thresholds) than either learners (p = 0.001) or nonlearners (p = 0.039). Mean performance in the 90% group did not differ by learning subgroup. We showed (p<0.02), using Monte-Carlo simulations (see Figure S1: Bootstrapping Analysis) that the group by subgroup interactions observed in our study did not result from a problem in data sampling.

IQ has been suggested to be causally linked to a form of pitch discrimination (auditory ‘inspection time’) [23], [24]. Non-verbal IQ (NVIQ) measured here also correlated with naïve DLFs (r = −0.53; p<0.001) and we have previously observed a significant correlation between Full-scale IQ and DLFs (e.g. [25]; unpublished data). However, IQ was found not to correlate with FD learning, either in this study (r = −0.10; p = 0.31) or in our previous work. We hypothesized that NVIQ might influence learning indirectly by playing a role in the utilization of feedback, predicting a significant interaction between NVIQ and learning subgroup. Figure 3D shows such an interaction (F(2,82) = 4.1, p = 0.020), supporting this hypothesis. Unlearners in the NF group had lower NVIQ than both other learning subgroups, which was not the case in the 90% group.

## Discussion

Our findings provide new insights into the role that both feedback and individual abilities play in learning. Using a large sample, we have shown that feedback can have multiple effects on learning the same task. Individual differences cannot be addressed when the sample is too small, a problem of many previous studies and possibly one of the root causes of the varied conclusions found in the literature [1]–[11]. A majority of the participants learned the task regardless of whether, or how much, positive feedback was provided. Whereas almost all participants either improved or maintained their performance when a small amount of feedback was provided (10% of trials), a substantial proportion failed to learn with either too much positive feedback (90%) or no feedback at all (NF). Participants who found the lack of feedback detrimental to learning had lower NVIQ than those who maintained or improved their performance. In contrast, learning by participants trained with 90% feedback was unrelated to NVIQ.

### Feedback is not used for trial-by-trial error correction

Various models derived from studying the behaviour of neural networks have been proposed to account for learning. Unsupervised learning (sometimes referred to as ‘Hebbian’ learning) models describe the process as being driven by bottom-up, feed-forward processing, independent of external feedback or reinforcement [26]. The learning in these models depends only on the stimulus, and ‘synaptic weights’ are updated based on the statistical distribution of the stimuli used for training the network. This purportedly occurs when the synaptic connections between neurons are strengthened through the repeated and consistent firing of a pre-synaptic neuron followed by a post-synaptic neuron [27]. In supervised learning models, feedback fills the role of an external ‘teacher’, driving learning in a top-down manner [28]. The external feedback generates the signals necessary to update the synaptic weights that ‘store’ the learned association between the stimulus and a correct response [29]. If the teacher signals a correct response, the relevant synaptic connections are strengthened. If it signals an error, no change occurs in synaptic weights in the network.

Applying these models we would make two different predictions for the present study. If the learning was unsupervised, we would predict that there would be no group differences in learning because unsupervised learning is independent of external feedback. In contrast, we found significant effects of external feedback on learning. Since the task was impossible the feedback was meaningless – it could not have been informative about correctness of listeners' responses, and could therefore not have been used as a teacher signal, ruling out supervised learning as an explanation for the results of our study. The same line of reasoning was followed in interpreting the results of feedback manipulation in a visual vernier acuity task [10], and the same conclusion was reached, suggesting the effect of feedback is a supramodal feature of perceptual learning.

‘Hybrid’ learning models have also been proposed to account for perceptual learning in previous studies. Most notably, a supervised Hebbian learning model was proposed to explain why feedback may be necessary in some cases but not others [17]. The success of unsupervised learning in this model depends on sufficiently high correlations between stimulus and response. Easy trials (or ‘exemplars’) are required to ‘jumpstart’ the learning process. The internal feedback generated by these easy trials serves the same function as would external feedback [2], [8], [30], [31]. When the task is too difficult and initial performance is not sufficiently above chance, Hebbian learning may be very slow to pick up on these weak correlations, and may even fail to do so. External feedback is then required to enhance the weights of correct decisions and bootstrap the learning process. Even if the small number of trials used to assess performance prior to training in our study were sufficient to bootstrap internally supervised learning (the ‘eureka effect’ [19]) we would expect no difference between training groups based on this model, since they all received the same pre-training Demo and Probe (Figure 1). Therefore, supervised Hebbian learning models can also be ruled out as an explanation for the current results.

The presence of easy trials may not be sufficient to generate internal feedback that can drive learning [16], but such feedback can be generated by the impossible training trials used in our study. Although not intuitively obvious, a mathematical derivation based on signal detection theory and the concept of internal neural noise [20], [21] showed that internal noise is sufficient to produce a percept of frequency differences between the identical tones [22]. Anecdotally, participants trained on identical tones with (random, 33%) feedback have complained that the feedback was “wrong”, as they were certain of their response. The feedback provided on this task is uncorrelated with the participants' responses (and hence internal feedback), a condition under which no learning was observed in a visual study [10]. If listeners use internally-generated feedback for trial-by-trial error correction, but cannot ignore the conflicting external feedback, changing the amount of external feedback should modulate its correlation with the internal signal, and hence the amount of improvement observed on the task. Assuming listeners are confident of their responses, learning should be greatest in conditions where response conflicts least with the external feedback. We would thus expect most learning in the NF condition, where there is no conflict, less learning in the 90% condition, where external feedback confirms their internal signal on 90% of trials, and very little learning in the 10% condition, where external feedback is largely uncorrelated with the response. This was not the case – we observed a U-shaped function in response to feedback manipulation where the NF and 90% groups showed no learning, on average.

A second type of hybrid model that nicely accounts for the results of feedback manipulation in vision [10] is a recurrent supervised model in which the feedback is not used as a teacher signal [32]. Instead, feedback (internal or external or both) is evaluated, and changes to synaptic weights due to unsupervised processes are stored only if the feedback evaluation is positive. This occurs via a selection process acting in a top-down fashion rather than propagating weight changes back through the entire network. Although this model does not require trial-by-trial feedback for learning to occur, it precludes learning when the internal and external feedback are uncorrelated because changes to weights cancel out over time due to the randomness of the feedback evaluation that results. This model would predict learning only in the no feedback condition in our study, since the external feedback would be uncorrelated with internal feedback in all feedback conditions (10%, 33% and 90%). This model therefore fails to account for our findings.

### Feedback can be used to monitor performance and enhance motivation

Like the recurrent supervised model discussed above [32], we propose feedback may be used to monitor performance rather than trial-by-trial error correction, by evaluating the expected performance in relation to the perceived difficulty of the task [33]. Learning was found to be suppressed when the external feedback conflicted with the internal evaluation of task performance in a visual experiment [34], suggesting that it is not the presence of feedback per se, but the appropriateness of the feedback that is key. Feedback is also known to modulate activity in executive and motivational circuits responsible for task performance [35]. Positive feedback can act as reward to encourage engagement with the task and reinforce the current learning strategy, while negative feedback (or the lack of positive feedback) can signal the need to switch to alternate strategies (e.g., by adjusting the decision criteria [17], [34]) or recruit additional attentional resources. We suggest that learning depends on motivation, which is modulated by the individual's subjective perception of how well they are performing.

People may become unmotivated when performing a training task for several reasons. Firstly, they can become bored and unmotivated when the trained task presents no challenge, such as when frequency discrimination is trained with a large frequency difference at which performance is at ceiling [18]. Secondly, motivation can be impaired when the task is perceived as being impossible, with no hope of success. Adding positive feedback on just 10% of trials had a profound effect on performance, leading to a ten-fold reduction in the number of listeners who showed unlearning compared to the no feedback condition. Listeners who may be unmotivated when receiving no external feedback to bolster confidence in their ability to perform the task may learn it when feedback is provided to confirm their decisions, even when this confirmation is minimal. Finally, as suggested above, reward may not motivate unless it is commensurate with the perception of how well one is performing under the circumstances (i.e., given task difficulty). This interpretation is supported by an EEG study that showed strong midbrain activation (which drives learning) only when participants perceived the feedback to be contingent on their responses [36].

Very little positive feedback (e.g. 10%) corresponds with the perceived difficulty of the task. The internal evaluation of the accuracy of performing the task can then be reinforced. On the other hand, there is a marked discrepancy between the internal evaluation of performance and external feedback when listeners who find the task very difficult receive a lot of positive feedback (90%). Listeners who unlearned when receiving 90% feedback may have perceived a mismatch between task difficulty and feedback, and may have stopped relying on external feedback, realising it didn't provide helpful information [34]. They may have “learned” to ignore the feedback during training, and continued to ignore it during the post-training DLF assessment, despite it being informative at that stage of the study. This would have resulted in their subsequent poorer performance relative to the pre-training assessment. In contrast, listeners who learn when provided 90% feedback may have adopted strategies that, albeit not leading to perceptual decision making based on the properties of the stimuli, may have convinced them that the feedback was contingent on their responses and could be used to improve their performance [37]. In debriefing after the experiment, one of the participants in the 90% group described the strategy used to do well on this task as choosing the same interval until the first incorrect response, then switching to responding consistently to another interval. Thus, feedback could either have been used to monitor performance and motivate even if the perceptual task was not actually performed, or it could discourage the listeners from paying attention to the sounds and simply press the button, resulting in either improvement or decrement in performance, as observed in the 90% group.

A recently published study [38] lends strong support to our views on feedback and motivation. After each training block of trials, fake feedback was provided that either followed the same rate of performance improvement as appropriate feedback, or corresponded to slower or faster learning. Feedback slightly above the appropriate level speeded up learning, whereas feedback slightly below appropriate had little effect on learning. Feedback that simulated deteriorating performance inhibited learning. These findings can be interpreted in terms of feedback driven motivation: when participants were led to believe they had done well their learning improved, and when the feedback was below expectations they showed signs of an ‘optimistic’, or ‘self-serving bias’, a concept used in educational psychology to describe a situation in which the feedback is undervalued [39]. When feedback signals declining performance, listeners eventually stop learning altogether, presumably because the optimistic bias does not offset the demotivating effect of feedback. Unlike the 90% feedback condition in our experiment, the differences between the “real”, expected performance and that signalled by the higher, fake feedback were small. Nonetheless, we have evidence for a similar trend in our data for the 33% group (Figure 2). Although 10% positive feedback may be more “realistic” with reference to the perceived difficulty of the task (only one participant in the 10% group realised that the feedback was given at less than chance level), 33% positive feedback yielded more learning. Our findings thus support the interpretation of learning being modulated by an optimistic bias. The fact that blocked [10], [38] and trial-by-trial feedback (our study) show similar effects on learning is further support for an interpretation that casts feedback in the role of monitoring performance rather than trial-by-trial error correction.

### IQ and self-monitoring

The group who received no feedback showed a similar lack of overall learning and a similar proportion of unlearners to the 90% feedback group, but the unlearners in the NF group were characterized by having poorer average performance thresholds and lower NVIQ. Problem-solving in everyday life does not always occur in conjunction with feedback about the solution or the correctness of the decision made. People who are able to apply themselves to challenging problem solving, whether they are given feedback on the correctness or level of their performance or not, are more likely to perform well on IQ tests, and in particular problem-solving tests such as the Matrix Reasoning subtest. The relationship between the ability to learn a task in the absence of feedback may therefore be related to the ability to monitor performance without external feedback, which may also be responsible for developing excellent problem-solving skills. This interpretation is compatible with studies of self-regulation in children. Self-regulation is defined as the ability to modify behaviour according to task demands in the context of the ability to delay gratification as well as the ability to ignore distracters when performing the task, and may thus be similar to the self-monitoring we describe here. Children with higher IQ show better self-regulation and are better able to sustain their superior performance on a difficult task than children with average IQ [40]. Note that the participants recruited for this study all had NVIQ within the normal range, but greater individual differences were found with no feedback, compared to a feedback condition, as in a previous visual learning experiment [10]. We hypothesize that more extreme group effects would be observed with no feedback if we recruited participants with a wider range of IQs.

Whereas most individuals can successfully monitor performance internally to employ the best strategy for the task and can therefore learn with or without feedback, others are not able to do so optimally and need external feedback to boost performance and to keep them motivated and on-task. The feedback can only fulfil this function when it matches the expectations derived from the perceived difficulty of the task. These findings highlight the importance of individual differences in understanding the cognitive processes that drive and support perceptual learning and, from an applied perspective, the need to take individual strengths or weaknesses into account when designing training programmes for a variety of applications.

## Materials and Methods

### Participants

One-hundred and six adults aged 18–40 were recruited from the University of Nottingham student population and from the general public and were paid an inconvenience allowance for their participation. All participants had normal hearing (pure-tone thresholds bilaterally ⇐20 dB HL across 0.5–4 kHz), except one participant who had mild hearing loss at 4 kHz in the right ear (35 dB HL) but was included in the study since pure-tone thresholds at all other frequencies (including those used in the experiment) were in the normal range (⇐20 dB HL).

### Ethics Statement

The research protocol was approved by the Nottingham Research Ethics Committee. Informed written consent was obtained from all participants.

### General Procedure

The study protocol consisted of a pre-test phase, a post-test phase and a training phase (Figure 1). All testing was completed within one session in a sound-attenuated booth. All phases were administered via “computer games” with a visual interface that cued sound presentation and provided visual feedback during testing of all participants and during training only for the groups that received feedback (see below). There was no time limit in which to respond, and the initiation of each trial was self-paced.

### Stimuli

Stimuli for both testing and training consisted of 100 ms tones (including 10 ms raised cosine ramps) presented with an inter-stimulus interval of 500 ms. In the test phases, standard tones had a frequency of 1000 Hz and target tones were varied adaptively. In the training phase, there was no frequency difference between standard and target tones so that all tones had a frequency of 1000 Hz. Stimuli were presented diotically using Sennheiser HD-25-1 headphones at 60 dB SPL.

### Pre- and Post-Training Phases

One frequency-discrimination (FD) threshold assessment of 30 trials (‘probe’; see [41]) was administered during each of the pre- and post-training phases. For each trial, listeners were presented with three intervals, two containing the standard tone (F), and the third, randomly-determined interval containing a higher-frequency tone (F+ΔF). Each interval was visually cued by an animated character, and listeners were instructed to indicate the interval they believed differed from the other two by touching the character that corresponded to the chosen interval on the touch-screen. Feedback was provided for correct responses by a brief animation of the correctly chosen character (jumping up and down). A 5-trial demonstration was administered before the pre-test probe to familiarize participants with task requirements. Three of these “demo” trials were ‘easy’ (ΔF = 50%), and two were impossible (ΔF = 0%). Listeners were instructed to guess when they could not hear a difference between the sounds. All participants correctly identified the target sounds for the ΔF = 50% demo trials.

The probes used an adaptive three-down, one-up staircase procedure, targeting 79.4% correct on the psychometric function [42]. ΔF varied adaptively according to the following rule: starting with ΔF = 50%, it was divided by 2 following every correct response until the first incorrect response. Thereafter, ΔF was divided by √2 after three correct responses, and multiplied by √2 after one incorrect response. Following two consecutive steps in the same direction (up or down), the step size was multiplied by √2. Difference limens for frequency (DLFs) were calculated as the 79.4% correct point on the logistic psychometric function fitted to the 30 trials in each probe using the Wichman and Hill procedure [43].

Listeners were allocated to one of three training groups based on their pre-test thresholds so as to match the groups as closely as possible on initial FD ability. A one-way analysis of variance (ANOVA) confirmed that the training groups were well matched on pre-test thresholds (F(2,91) = 0.016; p = 0.98).

### Training Phase

During the training phase, all tones were identical (ΔF = 0%) but listeners were instructed to perform the same discrimination task as that in the pre-test phase and choose the sound that was different. In two groups, positive feedback was provided on some trials such that listeners believed their response was correct. Listeners in the first training group (90%) received positive feedback on 90% of the trials, randomly picked by the software running the experiment. A second training group (10%) received positive feedback on 10% of the trials. Listeners in the final group (NF) received no feedback; they were informed prior to the first training block that they would not be receiving any feedback for that part of the session.

### Non-verbal IQ

The Matrix Reasoning subtest of the Weschler Abbreviated Scale of Intelligence (WASI [44]) was administered at the end of the post-test to assess non-verbal IQ. The test requires participants to choose the pattern (out of 5) that completes the matrix from which a section is missing. A one-way ANOVA confirmed that nonverbal IQ did not differ significantly between the groups (F(2,91) = 1.24; p = 0.29).

### Data Exclusions

Of the 106 participants recruited for this study 12 were excluded from statistical analysis because the psychometric function fitted to their pre- or post-test probe data had very shallow slopes (⇐0.10), which render the threshold estimates for these probes unreliable. Four participants were excluded from the 90% group (1 based on pre-test and 3 on post-test thresholds); three from the 10% group (all post-test); and five from the NF group (4 pre-test and 1 post-test), leaving 32 in the 90% group, and 31 each in the 10% and NF groups.

### Statistical analysis

FD threshold data (in Hz) were log-transformed, and all statistical analyses were carried out on the log-transformed data. A mixed-model analysis of variance (ANOVA) which allows for heterogeneity of variance was used to investigate the group data because the equality of variance assumption was not met by the data set (Levene's Test of equality of error variance: p = 0.038). Non-verbal IQ was included as a covariate in the model.

Learning subgroup analysis was carried out on the mean of pre- and post-training DLFs rather than the learning index (difference between pre- and post-training DLFs) because the former is statistically independent from the subgroup factor whereas the latter is not (i.e. the learning index is correlated with the pre-training DLF but (almost) uncorrelated with the mean of pre- and post-training DLFs).

The figures include an additional group (33%) which was collected in a previous study with 33% positive feedback ([18]; ‘Constant 0 Hz’ group at the mid-training threshold assessment). These data are displayed for comparison only and are not included in the statistical analysis due to differences between the two studies: there were 400 trials of training instead of the 600 used here, and a maximum likelihood algorithm was used to assess FD thresholds rather than a staircase.

## Supporting Information

Figure S1 Bootstrapping Analysis. Monte-Carlo simulations were conducted to confirm the main finding of differences between learning subgroups in the manuscript did not result from a problem in data sampling.
(0.03 MB DOC)

## References

[1] Zwislocki J, Maire F, Feldman AS, Rubin H (1958). On the effect of practice and motivation on the threshold of audibility.. J Acoust Soc Am.

[2] Lukaszewski JS, Elliott DN (1962). Auditory threshold as a function of forced-choice technique, feedback, and motivation.. J Acoust Soc Am.

[3] Campbell RA, Small AM (1963). Effect of practice and feedback on frequency discrimination.. J Acoust Soc Am.

[4] McKee SP, Westheimer G (1978). Improvement in vernier acuity with practice.. Percept Psychophys.

[5] Ball K, Sekuler R (1987). Direction-specific improvement in motion discrimination.. Vision Res.

[6] Karni A, Sagi D (1991). Where practice makes perfect in texture-discrimination: Evidence for primary visual-cortex plasticity.. Proc Natl Acad Sci U S A.

[7] Shiu LP, Pashler H (1992). Improvement in line orientation discrimination is retinally local but dependent on cognitive set.. Percept Psychophys.

[8] Fahle M, Edelman S (1993). Long-term learning in vernier acuity: Effects of stimulus orientation, range and of feedback.. Vision Res.

[9] Crist RE, Kapadia MK, Westheimer G, Gilbert CD (1997). Perceptual learning of spatial localization: Specificity for orientation, position, and context.. J Neurophysiol.

[10] Herzog MH, Fahle M (1997). The role of feedback in learning a vernier discrimination task.. Vision Res.

[11] Fahle M, Edelman S, Poggio T (1995). Fast perceptual learning in hyperacuity.. Vision Res.

[12] Tallal P, Miller SL, Bedi G, Byma G, Wang X (1996). Language comprehension in language-learning impaired children improved with acoustically modified speech.. Science.

[13] Merzenich MM, Jenkins WM, Johnston P, Schreiner C, Miller SL (1996). Temporal processing deficits of language-learning impaired children ameliorated by training.. Science.

[14] Polat U, Ma-Naim T, Belkint M, Sagi D (2004). Improving vision in adult amblyopia by perceptual learning.. Proc Natl Acad Sci U S A.

[15] Mahncke HW, Connor BB, Appelman J, Ahsanuddin ON, Hardy JL (2006). Memory enhancement in healthy older adults using a brain plasticity-based training program: A randomized, controlled study.. Proc Natl Acad Sci U S A.

[16] Seitz AR, Nanez JE, Holloway S, Tsushima Y, Watanabe T (2006). Two cases requiring external reinforcement in perceptual learning.. J Vis.

[17] Petrov AA, Dosher BA, Lu ZL (2006). Perceptual learning without feedback in non-stationary contexts: Data and model.. Vision Res.

[18] Amitay S, Irwin A, Moore DR (2006). Discrimination learning induced by training with identical stimuli.. Nat Neurosci.

[19] Ahissar M, Hochstein S (1997). Task difficulty and the specificity of perceptual learning.. Nature.

[20] Swets JA (1964). Signal detection and recognition by human observers.

[21] Green DM, Swets JA (1966). Signal detection theory and psychophysics.

[22] Micheyl C, McDermott JH, Oxenham AJ (2009). Sensory noise explains auditory frequency discrimination learning induced by training with identical stimuli.. Percept Psychophys.

[23] Deary IJ (1994). Intelligence and auditory-discrimination: Separating processing speed and fidelity of stimulus representation.. Intelligence.

[24] Deary IJ (1995). Auditory inspection time and intelligence: What is the direction of causation.. Dev Psychol.

[25] Amitay S, Hawkey DJC, Moore DR (2005). Auditory frequency discrimination learning is affected by stimulus variability.. Percept Psychophys.

[26] Hinton GE, Sejnowski TJ (1999). Unsupervised learning: Foundations of neural computation.

[27] Hebb DO (1949). The organization of behavior.

[28] Knudsen EI (1994). Supervised learning in the brain.. J Neurosci.

[29] Reynolds JNJ, Wickens JR (2002). Dopamine-dependent plasticity of corticostriatal synapses.. Neural Netw.

[30] Seitz A, Watanabe T (2005). A unified model for perceptual learning.. Trends Cogn Sci.

[31] Holroyd CB, Nieuwenhuis S, Yeung N, Nystrom L, Mars RB (2004). Dorsal anterior cingulate cortex shows fMRI response to internal and external error signals.. Nat Neurosci.

[32] Herzog MH, Fahle M (1998). Modeling perceptual learning: Difficulties and how they can be overcome.. Biol Cybern.

[33] Ferdinand NK, Mecklinger A, Kray J (2008). Error and deviance processing in implicit and explicit sequence learning.. J Cogn Neurosci.

[34] Herzog MH, Fahle M (1999). Effects of biased feedback on learning and deciding in a vernier discrimination task.. Vision Res.

[35] Seger CA (2008). How do the basal ganglia contribute to categorization? Their roles in generalization, response selection, and learning via feedback.. Neurosci Biobehav Rev.

[36] Tricomi EM, Delgado MR, Fiez JA (2004). Modulation of caudate activity by action contingency.. Neuron.

[37] Holroyd CB, Larsen JT, Cohen JD (2004). Context dependence of the event-related brain potential associated with reward and punishment.. Psychophysiology.

[38] Shibata K, Yamagishi N, Ishii S, Kawato M (2009). Boosting perceptual learning by fake feedback.. Vision Res.

[39] Alloy LB, Abramson LY (1979). Judgment of contingency in depressed and nondepressed students: Sadder but wiser?. J Exp Psychol Gen.

[40] Calero MD, Garcia-Martin MB, Jimenez MI, Kazen M, Araque A (2007). Self-regulation advantage for high-IQ children: Findings from a research study.. Learning and Individual Differences.

[41] Amitay S, Irwin A, Hawkey DJC, Cowan JA, Moore DR (2006). A comparison of adaptive procedures for rapid and reliable threshold assessment and training in naive listeners.. J Acoust Soc Am.

[42] Levitt H (1971). Transformed up-down methods in psychoacoustics.. J Acoust Soc Am.

[43] Wichmann FA, Hill NJ (2001). The psychometric function: I. Fitting, sampling, and goodness of fit.. Percept Psychophys.

[44] Psychological Corporation (1999). WASI: Wechsler Abbreviated Scale of Intelligence.

