# Supplementary material for: Motivation and Intelligence Drive Auditory Perceptual Learning
Source: PLoS One. 2010 Mar 23;5(3):e9816. doi: 10.1371/journal.pone.0009816 (PMC2843743; doi:10.1371/journal.pone.0009816)
Supplement: Figure S1 — Bootstrapping Analysis. Monte-Carlo simulations were conducted to confirm the main finding of differences between learning subgroups in the manuscript did not result from a problem in data sampling. (0.03 MB DOC) [file pone.0009816.s001.doc]

**Motivation and Intelligence Drive Auditory Perceptual Learning**

**Sygal Amitay, Lorna Halliday, Jenny Taylor, Ediz Sohoglu, & David R. Moore**

**Supplementary Material – Bootstrapping Analysis**

In order to show that individuals in the 90% group and No feedback (NF) groups are differentially affected by the feedback manipulation despite both groups showing similar mean learning, we subjected the data to a bootstrapping analysis using a Monte-Carlo simulation. We tested the null hypothesis that the two groups are sampled from the same listener population, that is, that the experimental manipulation affects all listeners in the same manner. We first pooled together all listeners from both groups, and randomly resampled the pre- and post-training DLFs, and then randomly regrouped the listeners into two groups, keeping the original group numbers. Next we calculated learning as the difference between assigned “pre-training“ and “post-training” DLFs, and divided listeners into learning subgroups as with the original data, and performed separate one-way analyses of variance (ANOVA) on the average performance DLFs for each group by learning subgroup. The distributions of F-values associated with 10,000 iterations of this process are shown in Figure S1 below, with the F-values associated with the original data set marked as red lines. Over 90% of all F-values are higher than the F-value of the ANOVA associated with the real data set for the 90% group confirming that there is no difference in average performance between learning subgroups. Less than 2% of all F-values are higher than that associated with the real data set for the NF group, suggesting the difference between learning subgroups is highly significant. These results confirm that the difference between the 90% and NF groups in average performance DLFs among learning subgroups are the result of the experimental manipulation and not a problem in data sampling or measurement error.

**Figure S1.** Distribution of F-values for ANOVAs across learning subgroups for the 90% group (top panel) and NF group (bottom panel).
